# Exfoliation of the Mucous Membrane of the Uterus; Treatment by Canula in the Os Uteri, Etc.

**Published:** 1855-08

**Authors:** 


*Exfoliation of the Mucous Membrane of the Uterus; treatment by canula in the os uteri, etc.* Under the care of Dr. TYLER SMITH.

Cases of what are styled "membranous menstruation," with the excessive pain attending this affection, are not uncommon in general practice, yet it is only lately that they have come to be properly understood. The older pathologists, as Baillie and Wm. Hunter, believed dysmenorrhœa to depend on an inflammatory condition of the uterus at the period of menstruation, and that even in the virgin uterus an organized substance, resembling the decidua, was formed by the lining membrane, and thrown off, when a small organized mass, the shape of the cavity of the uterus, was passed. Whether in the married or unmarried condition, it was known in common language as a "mole."

Dysmenorrhœa sometimes takes place from the very commencement of menstrual life, and there is good reason for believing that it depends on the small size or strictured condition of the os uteri. The menstrual fluid, after it is formed or while forming, cannot readily escape; distention of the organ speedily follows, which, by exciting the contraction of the uterine fibres, produces pain almost simulating that of labor. Even the action of the abdominal muscles is called into play, and many cases of what are termed "spurious pregancy" may be very possibly explained in this manner. It is believed, too, that women thus affected rarely, if ever, conceive or bear children, the normal healthy function of the uterus being interfered with, as well as the woman's health reduced by the constant suffering and pain. Of all the means of cure hitherto tried, dilatation of the canal of the cervix uteri seems to be the best, to which more recently has been added the introduction of a silver canula, as tried by Dr. Tyler Smith.

When in 1846 Professor Simpson, of Edinburgh, announced that the membrane expelled in certain cases of dysmenorrhœa was not simply a fibrinous or inflammatory exudation, the result of some change in this organ, but consisted rather of "exfoliation or detachment of the mucous membrane of the uterus itself," the obstetric world, according to Dr. Tyler Smith, as he explained to his class, was somewhat startled from its propriety, and ever since that time, when the matter has been referred to, it has generally been with strong expressions of doubt and incredulity. Obstetric practice has been hitherto rather undeservedly under a cloud in London, and few opportunities have been afforded to test the matter. We are not aware that any researches bearing directly upon Dr. Simpson's discovery have been published in London, so that the following case, abridged from the notes taken in St. Mary's may be instructive:—

M. D——, aged thirty-two, twice married, but without children, was admitted April 20th, suffering from dysmenorrhœa and the discharge of membranous matter at each monthly period. The history of the case, as taken by Dr. Vernon, was as follows:—She was first married at the age of sixteen, and before this time had menstruated healthily. After marriage she began to suffer from menorrhagia, which she supposed to have been caused by excessive intercourse. This went on, and ended in membranous menstruation, accompanied by profuse sanguineous discharge. She continued to suffer in this way during the lifetime of her first husband; but seven years after her marriage she became a widow. She now slowly recovered her health menstruation becoming less painful, and the dysmenorrhœal membrane disappeared altogether. She remained unmarried for several years, but married a second time about two years ago. Six months after this marriage, she began to suffer pain again during the menstrual periods, and the characteristic membranous shreds reappeared in the discharge. She now for the first time had internal hæmorrhoids, with fissure of the rectum; blood was occasionally passed by stool; and defecation, except under the influence of laxatives, caused great pain; micturition was also painful. In the intervals of menstruation she complained of leucorrhœa, the discharge being sometimes of a brownish color. Occasionally severe pruritus was present. She had been a patient at several public institutions without obtaining material relief.

At the end of last year, when she first became an out-patient at St. Mary's Hospital, her health was much broken by menstrual losses, the dysmenorrhœal pain and the leuconhœal discharge. At this time the membranous matter came away in flakes of considerable size, the largest generally passing on the first days of menstruation. The greatest pain was suffered on the first day of the discharge and the two or three days preceding its appearance. On examination, the uterus was of the natural size, and the only thing tobe remarked was a very contracted state of the os uteri, scarcely admitting the end of a probe into the canal of the cervix. Various remedies were used with the effect of moderating the leucorrhœal discharge and improving the general health. The os uteri was on several occasions dilated mechanically, but it was soon found to return to its former state of contraction. On one occasion Dr. Smith introduced into the cervix uteri a small silver canula about three quarters of an inch long, with a view to its remaining in the canal during a catamenial period. She menstruated while wearing this instrument, the discharge coming away freely in a granular instead of membranous form, and she suffered much less pain than usual. She herself stated that she had not been so free from pain at any month since the time the disorder reappeared after her second marriage. Some days after menstruation had ceased, this canula was taken out, and at the next two or three monthly periods the pain and membranous discharge returned as before. Dr. Tyler Smith now admitted her into the hospital, with the view of watching the case more closely, and ascertaining, if possible, the exact nature of the discharge. Shortly after admission menstruation took place, and the membranous flakes were carefully collected and examined with a lens and by the aid of the microscope. The largest membranous flakes exceeded the size of a shilling, and were about the eighth of an inch in thickness. When examined in water, they presented on one side a smooth surface, and on the other side the surface was flocculent and irregular. A vertical section showed the membrane to consist of a fibroid layer, in which was imbedded an abundant cell-formation, made up of multitudinous nuclei and celloid particles. From the free or smooth surface, numerous villi projected, while from the flocculent surface, long tubular glands could be seen passing downwards. These glands had a basement membrane, surrounded by an outer thin coating of nuclei and fibroid tissue, and were lined with epithelium. The tubes of the glands were full of cylinder epithelium, nuclei, and granular matter. The tubular glands could be seen with the naked eye, and, with the villi of the surface, were characteristic of the uterine mucous membrane. There appeared to be no doubt that the membranous flakes were the mucous membrane of the fundus uteri, cast off *in toto.*

This case, then, strongly supports the pathological views of Dr. Simpson. An intelligent young German physician, Dr. Spiegelberg, of Gottingen, who saw this case with Dr. Smith, informed us that the celebrated Virchow, of Wurzburgh, and Professor Heschl, of Olmutz, formerly senior assistant to Rokitansky at Vienna, had examined these exfoliations, and found them also to consist of detached mucous membrane.

On the approach of the next monthly period, after this examination of the membranous formation, the silver canula was again introduced, with the effect of diminishing the pain as before, and the discharge also contained much less flaky matter. The tube was allowed to remain *in situ,* and a second period has since been passed with marked diminution of pain, and alteration in the character of the discharge.

We have given an account of the present case because of its intrinsic interest, and to note the relief afforded by keeping the os uteri open with the canula. It remains to be seen how far the benefit derived in this way will proceed, but the use of such a means is certainly rational in a form of disease which obstinately resists ordinary modes of treatment. In other cases of membranous menstruation treated at St. Mary's, Dr. Smith has tried local abstraction of blood, counter-irritation, mild mercurialization, iodism, tartar emetic, (given at the periods so as to keep up constant nausea,) opiates, preparations of steel, cubebs, &c. &c., without arriving at any certain means of checking this troublesome disease. We ought not to omit to mention that in the case now under comment, full doses of camphor lupulin had been given with a view to their antiaphrodisaic effects.—*London Lancet.*

